# From Solution Traps to Solution Patchwork: Easing Tensions in Designing Digital Health in the Global Context

**DOI:** 10.2196/82214

**Published:** 2026-07-13

**Authors:** Dario Staehelin, Gianluca Miscione, Mateusz Dolata

**Affiliations:** 1 School of Management, Eastern Switzerland University of Applied Sciences, Rosenbergstrasse 59, St. Gallen, 9001, Switzerland, 41 58 257 31 06; 2 Department of Informatics, University of Zurich, Zurich, Switzerland; 3 Smurfit Graduate Business School, University College Dublin, Carysfort Avenue, Belfield, Dublin, D04 V1W8, Ireland; 4 Institute of Business Information Technology, School of Management, ZHAW Zurich University of Applied Sciences, Winterthur, Zurich, Switzerland

**Keywords:** phenomenon-based problematization, digital health, context-centric problem solving, design science research, medical informatics, institutional logics

## Abstract

Two recently published viewpoint articles by *JMIR Publications* highlighted the 2 faces of medical informatics research. On the one hand, they spotlight the significant advances digital technologies bring to health management and delivery. Over the last decade, technological advances have transformed health care worldwide. Consumers have much broader access to digital health tools, gathering an unprecedented amount of data that can be used to gain personalized insights. Digital technologies support medical professionals in clinical and administrative work like never before. On the other hand, these articles emphasize the persisting divide between the potential of these technologies and their integration into everyday practice. Substantial barriers remain that often hinder the broader adoption of digital health tools. In this viewpoint article, we offer a pragmatic perspective on a problem often overlooked in medical informatics: the incompatibility of coexisting solutions in complex sociotechnical contexts. It problematizes underlying assumptions in medical informatics–oriented and design-oriented research approaches, such as design science research, more precisely by conceptualizing the “solution trap.” The solution trap refers to situations in which designers introduce a new solution without recognizing existing ones, creating tensions among coexisting sociotechnical practices. We emphasize the need for a nuanced understanding of context unevenness and propose solution patchwork as a coordination approach to evade the solution trap. Solution patchwork describes an approach in which designers integrate new artifacts into established practices and institutional logics to ensure compatibility across the broader sociotechnical system. The solution patchwork sensitizes medical informatics researchers and designers to navigate the problem and solution spaces in health systems globally.

## Introduction and Key Message

### Motivation

Digital health failures often stem less from “no solution” and more from clashing, coexisting solutions in uneven contexts. Sociotechnical misfit explains digital health failures better than the inherent properties of technology alone [1,2]. Digital technologies can build resilience, but the COVID-19 immunization setbacks showed how a technology-context disconnect—world-class vaccines but uneven acceptance and coverage—can undermine that potential. Against this background, we argue that incompatibilities between coexisting solutions in complex sociotechnical systems often stem not from misaligned stakeholder goals but from contextual unevenness and diverse institutional logics. Digital health is shifting health care from provider-centered to consumer-driven models, empowering consumers, assisting health care professionals, and bridging infrastructure gaps [3]. However, fragmentation within the health care innovation ecosystem remains a major barrier to translating research insights into practice [4].

Once the COVID-19 pandemic spread globally, vaccines were widely expected to contain the virus and restore normality. The assumption was that once vaccines were available, administering them broadly would end the pandemic. As we now know, vaccine acceptance was far lower than expected. Reasons included mistrust in vaccine science, conspiracy theories amplified by social media algorithms, lack of education, and political opportunism [5]. In many countries, governments struggled to counteract vaccine hesitancy. They had to work hard to reverse citizens’ drift away from what had seemed an obvious course of action only months earlier. This discrepancy between expectations and behavior is widespread and more common than often assumed. Digitalization may exacerbate this phenomenon by bringing people, organizations, and societies across diverse contexts into closer interaction [6].

The divergences exhibited by different social groups with their own values and interests [7] can be attributed to distinct institutional logics that shape their orientations and behaviors [8,9]. This paper supports this argument by showing that the organizational contexts in which people use technologies are often shaped by *conflicting* logics that hinder coordination and implementation. Furthermore, it identifies and discusses the problem of coexisting solutions, which we refer to as the “solution trap.”

As pressing issues such as global health equity increase the need for cooperation, we emphasize the importance of addressing the solution trap to improve coordination and implementation, thereby supporting sustainable resilience. Researchers and designers, often driven by academic rigor rather than an awareness of the diverse contexts in which global IT operates, may apply theories or methods that result in a reductionist view of the local context. This often creates mismatches between designed interventions and their broader implementation contexts, undermining the outcomes of globally deployed efforts.

The lack of culture-sensitive and context-specific theorizing in research has been noted earlier, resulting in calls for a new generation of theories [10-12]. Design research has taken up this line of reasoning but has focused mainly on adapting user interfaces to make software usable across cultural boundaries [13-15]. However, we argue that the issue runs deeper. We argue that designers and researchers often overlook or disregard existing solutions within the target context for 2 main reasons. First, these solutions may be deeply embedded in the local environment, making them implicit and difficult for external observers to identify. Second, they may conflict with the theoretical perspective guiding the design researcher’s focus, which often assumes that theories, methods, and solutions are transferable across settings. As a result, valuable local insights and approaches are frequently missed or dismissed, resulting in the notion of a void that needs to be filled. Identifying, exploring, problematizing, and, where appropriate, augmenting existing solutions in relation to the local context and stakeholder needs could lead to more sustainable interventions.

The current situation can be compared to wearing “blinders,” limiting researchers’ perspectives and leading to a focus on technical systems operating in a vacuum, separate from the social realities they are ultimately intended to address. However, this vacuum disappears once the intervention is implemented, as it inevitably becomes part of a complex sociotechnical problem space. It becomes exposed to the specific context, pre-existing processes, and users’ changing beliefs, which can significantly influence its success or failure. The COVID-19 vaccine illustrates this: the technical system can be transferred globally, but it encounters different local social systems, resulting in varying levels of acceptance or rejection.

### Research Objective

This viewpoint aims to elucidate how incompatibilities between coexisting solutions in complex sociotechnical systems often arise not from conflicting stakeholder goals but from contextual unevenness and diverse institutional logics. We problematize implicit assumptions in problem-solving approaches commonly used in medical informatics, particularly design science research (DSR), to better understand these challenges. Drawing on global digital health examples from our own experience, we aim to (1) conceptualize the solution trap that arises when solution-driven efforts overlook contextual heterogeneity and (2) propose solution patchwork as a complementary approach for navigating diverse contexts within complex systems. This viewpoint is aimed at designers and researchers in medical informatics alike. It helps designers understand the context for which they design and add carefully to pre-existing patterns. For researchers, this viewpoint offers a novel way that inspires context-adapted problematization, challenging previous assumptions.

## Conceptual Boundaries and Definition of the “Solution Trap”

### Overview

Sociotechnical, institutional, and practice theories have broadened attention in research and practice to include the actual context of digital health solutions, their dynamics, and their evolution beyond design intentions [6-8]. Digital health interventions can be viewed as sociotechnical phenomena in which technical artifacts, professional practices, and local meanings coevolve, rendering solutions A and B mutually incompatible despite each being technically sound [16-18]. Chatterjee and Davison [19] argue that gap-spotting (ie, reporting a shortcoming of a technology) is insufficient and that research should problematize taken-for-granted assumptions, foreground context, and relate to local concepts when appropriate, even during the problem space phase.

Against this background, we see the solution trap as a phenomenon resulting from a narrow definition of the problem space in engineering and design projects. Designers and researchers construct solution traps by specifying a problem as a *lack of solution* rather than acknowledging and problematizing the solutions that already exist in the target context. We argue that the real issue in many IT-related engineering and design projects is not a lack of solutions but their abundance and incompatibility, which only increase as such projects add more solutions. Therefore, we define the “solution trap” as a sociotechnical accommodation according to which an artifact’s technical affordances, the organizational context, and the local meanings attached by user groups coproduce incompatibility with neighboring solutions. Evading such traps requires local bricolage and negotiated patchworks over universal solutionism.

### Design Science Research

The health care sector uses various technological and sociotechnical solutions to tackle persistent issues, such as staff shortages, treatment adherence, and limited medication access. While design and engineering approaches dominate this field, they often struggle to scale across different contexts [20]. In medical informatics, DSR and similar methods are frequently used and considered appropriate for health care [21]. DSR is rooted in the science of the artificial [22,23], Heideggerian philosophy [24], and early perspectives of engineering as a tool for scientific inquiry [25]. As a problem-solving approach, it uses engineering solutions to meet specific stakeholder needs [24]. However, a DSR project aims to do more than create a single solution; its value lies in deepening the understanding of a problem class and guiding future interventions [26,27]. Therefore, thoroughly grasping the problem and its broader consequences is essential in DSR [24].

All major DSR process models, methods, and frameworks begin with understanding and formulating the problem [27-30]. Experts agree that designing an intervention or artifact occurs in parallel with learning about the problem, creating a reciprocal relationship between the two. Over time, problem understanding and the solution converge: problem framing becomes more specific as the vision for the artifact materializes [31]. Ultimately, the proposed solution is evaluated against project goals derived from stakeholder needs [32]. Ideally, the artifact solves the problem, meets these requirements, and generates broader knowledge.

Recent debate on defining problems and problem spaces [24,27,31,33,34] has led to the idea of problem-space exploration as a stand-alone contribution [35]. Much of this discussion focuses on what defines a problem space and its boundaries. While the literature identifies broad categories such as stakeholders [24], organizations [34], and contexts [36], few studies attempt to unpack these categories deeply. When they do, the resulting framings often remain recursive [34]. Nevertheless, there is consensus that iterative exploration of the problem space is essential.

The parallel development of problem and solution space understanding suggests that researchers undergo a sensemaking process. Sensemaking involves 3 steps: establishing provisional meanings about the environment, acting on them, and revising those meanings when contradictory cues arise [37]. However, humans often prefer confirming evidence over contradictory data, which can delay necessary revisions [38]. To make sensemaking more effective, actors must remain mindful and actively seek additional evidence that prompts revision [39]. Since it is difficult to know where to look for such evidence, researchers may need guidance to consider the entire environment rather than being preselective. For design researchers, this means that solid problem understanding requires moving beyond what is readily apparent to engage deeply and holistically with the situation.

This broad and deep understanding of the problem space is often lacking, particularly in design-oriented publications [31]. These works tend to describe and evaluate solutions rather than reflect on how the problem space evolved during the process. They often fail to assess whether the final framing remains adequate and relevant to stakeholders and their contexts. This may explain why many DSR artifacts see little use in real-world settings. We argue that these issues stem from an oversimplified understanding of both the problem and the stakeholder needs addressed by the resulting interventions.

### Medical Informatics

The COVID-19 pandemic has underscored the critical role of medical informatics in public health responses. The rapid adoption of telehealth services during the pandemic demonstrated the potential of digital health tools to facilitate health care delivery in times of crisis [3,40,41]. Medical informatics is a relatively young, multidisciplinary research field that has recently expanded its scope under the broader name of “digital health.” It aims to improve health by using information technology to develop digital tools that address health issues worldwide [42]. Consequently, research has a strong focus on designing, deploying, and evaluating digital health tools. The field has gained significant traction in recent years, especially under the umbrella of “digital health,” which includes a wide range of technologies for improving health outcomes through information technology. The primary objective of medical informatics is to leverage these technologies to optimize health care delivery, enhance patient outcomes, and promote health equity across diverse populations [17,43,44]. Medical informatics has evolved rapidly through advances in digital technologies, such as artificial intelligence (AI), big data analytics, and mobile health apps [45]. These technologies have enabled the development of innovative digital health tools that promise to address pressing global health issues. For instance, AI algorithms increasingly assist clinicians in diagnosis by analyzing complex imaging data, thereby improving decision-making and patient care [16,46]. Big data analytics also helps identify trends and patterns that can inform public health strategies and interventions [47,48].

Recent medical informatics research can be grouped into 2 main domains: data-centered and human-centered studies [49,50]. Data-centered studies focus on technical aspects of health informatics, such as algorithms and data management systems that improve clinical decision-making and patient outcomes [51,52]. For example, studies show that AI can improve diagnostic accuracy by analyzing imaging data more effectively than traditional methods [53,54]. Human-centered studies, by contrast, emphasize user experience and the social implications of digital health tools. These studies explore how digital health technologies can be designed to meet the needs of diverse populations, particularly in underserved areas [55,56].

One notable application of medical informatics is telemedicine, which has proven vital in bridging the gap between health care providers and patients, especially in rural and remote areas. Telemedicine initiatives have been implemented in various contexts, such as the upper Amazon [18], where digital health tools have facilitated communication between citizens and health care professionals over vast distances [57,58]. Community health workers (CHWs) have also increasingly used digital tools to deliver essential health services, including maternal care, thereby improving health care access and equity [59,60]. Community-based health care enables lay people, such as CHWs, to provide critical health services, including maternal care [61]. In community-based health care, digital tools enhance the effectiveness of CHWs in addressing the medical staff shortage by providing basic, high-quality health services to their community [62]. However, these tools often neglect the users’ preferences [63].

Despite these advances, researchers have identified several challenges in designing and implementing digital health tools. A significant issue is the tendency for these tools to overlook user preferences and contextual factors that influence their effectiveness. For instance, while user-centered design approaches, such as DSR, aim to create artifacts that solve real-world problems, they often fail to account for the complexities of the environments in which these tools are deployed [64,65]. This context blindness can lead to suboptimal outcomes, as evidenced by the limited impact of many rigorously designed digital health interventions in real-world settings [66,67]. Moreover, integrating digital health tools into community-based health care systems has highlighted the need for comprehensive training and support for CHWs. Studies have shown that equipping CHWs with mobile health technologies can significantly enhance their ability to deliver care, particularly in low- and middle-income countries [68,69]. However, successful implementation requires not only technical training but also an understanding of the sociocultural dynamics that shape health behaviors within communities [70,71]. Medical informatics is prone to generating adverse effects if it falls into the solution trap. For example, dissonance and overwhelm caused by new interventions might lead intended users to abandon treatment altogether, especially when an old set of solutions suggests one course of action and a new one suggests another. We, therefore, argue that medical informatics requires especially careful contextualization.

In conclusion, maximizing the impact of these tools requires a holistic approach that considers user needs, health care system complexity, and the sociocultural factors that shape health behaviors. We argue that the ambiguous global outcomes of medical informatics stem from its single-minded problem-solving orientation. As outlined above, design methods aim to address stakeholder-relevant real-world problems with new artifacts. However, these methods often show context blindness that undermines the impact of designed artifacts in real-world deployment. How many scientifically rigorous artifacts based on well-established theories and approaches actually make an impact for users in their real contexts? Regretfully, not many [72].

## Empirical Illustrations

### Overview

In our research, we encountered multiple instances where translocal solutions created tensions when implemented locally. These accounts led us to conceptualize the solution trap. A solution trap occurs when designers define a problem as the “absence of a solution,” overlooking existing local solutions and the institutional logics that sustain them. New artifacts create coexistence conflicts, leading to rejection, workarounds, or brittle adoption. In this section, we first specify the solution trap concept and then illustrate it with examples from our research projects. We use a sociotechnical systems perspective, emphasizing the multidirectional relationships among technology, people, and their context [73].

### The Solution Trap

Most health settings host multiple, partially overlapping solutions rather than awaiting a fix. Adding one more without reconciling existing solutions creates coexistence conflicts: a solution trap. The solution trap is a misconstruction of the problem space in engineering and research projects. Designers and researchers perceive application contexts as incomplete and specify problems as *lacking solutions*. For instance, limited access to clinicians is framed as a lack of personnel or communication channels. Existing practice is deemed insufficient, requiring significant change or replacement. The reasons and origins of current practices remain uncovered. Solutions traps arising from such reductionism might lead to conflicts and unexpected, if not undesired, behaviors among stakeholders.

DSR and medical informatics researchers aim to address problems by designing solutions that are deployed in specific contexts. Often, deployment results in a combination of established and new solutions. This combination may appear successful initially. For example, telemedicine extends the health system’s reach to rural areas. It promotes the biomedical approach to diagnosing and treating patients to improve health care. Telemedicine enables remote diagnosis, helping patients receive the care they need. However, closer examination might reveal that these approaches can fall into the solution trap. Various stakeholders (eg, local clinicians and subject-matter experts) introduce their own problem-solving approaches.

A new solution inevitably interacts with existing solutions, risking the creation of new problems. The issue is coexistence and tensions arising from partially incompatible solutions, not a lack of solutions. This incompatibility could have 2 consequences. First, recipients may reject new interventions in favor of established practice. Rejection occurs when designer-intended behavior does not align with their context. Consequently, the new artifact is not adopted, and the intervention fails. Alternatively, recipients may adopt the solution to align with their current behaviors. User-centered approaches, such as DSR, aim to mitigate failures by exploring the problem space beforehand. Still, they provide little guidance on considering both existing and desired practices.

#### Illustration 1: A Stability Problem

A digital health project aimed to implement and study telemedicine to improve health conditions in the Peruvian Amazon. The idea was that telemedicine makes knowledge more accessible to rural health care practitioners. The system used a biomedical treatment approach designed to support accurate diagnoses from patient data. However, too many patients were diagnosed with malaria, even when symptoms and data suggested otherwise. This mismatch was particularly puzzling because the diagnosticians were trained doctors. Hence, a lack of knowledge could be ruled out as the cause of this problem.

In retrospect, doctors acted to maintain health system stability. They diagnosed based on available medication (ie, for malaria), avoiding diagnoses requiring unavailable resources. In this illustration, malaria medication is abundant, while other medications are scarce. Availability of medicines depends on many factors, including what is assumed to be a significant public health problem, like malaria. Understandably, funding comes with expectations of use. If the supply is not flexible enough, meeting expectations and securing insurance for future funding require not wasting what is available. This contrasts with contexts where biomedicine is well-established and well-resourced. In such contexts, doctors assess their patients’ symptoms and gather data to support their diagnoses. The treatment is selected based on diagnosis, not treatment availability. In the illustration above, it appears that medicine is practiced backward: resource availability circumscribes the range of possible diagnoses.

With the introduction of telemedicine, contrasting institutional logics collided. The designers fell into the solution trap, assuming insufficient knowledge distribution in the Peruvian Amazon. However, the problem was not the lack of knowledge but rather the actual options available to health personnel and patients. Established local practices foster the stability of the health care system, whereas the translocal intervention is designed to facilitate biomedicine through an information system. The solution trap creates a conflict of interest, forcing doctors to choose between correct diagnoses and available cures. Favoring stability over biomedical procedures may not seem ethically correct. However, malaria treatments also prevent malaria, increasing overall population prophylaxis without deliberate harm. Doctors may prioritize the overall health system for the greater good over individual cases. In summary, local logic (stability under scarcity) overruled translocal biomedical logic (diagnose-then-treat), causing the telemedicine “patch” to collide with medication availability and funding expectations.

This situation may lead to cascading effects as unintended consequences of the solution trap. Patients may feel dissatisfied, facing problems without a proper solution. Dissatisfied patients may start to distrust the health care system or public services. Another consequence could be black markets for scarce medication. Patients may buy medication from dubious sources, paying hefty markups or receiving contaminated or counterfeit drugs.

#### Illustration 2: An Accountability Problem

An international project implemented digital health tools to support public health, transitioning from legacy to open-source software. The new software promised to reduce vendor dependency, cut costs, and improve local capacity to adapt to the variety of implementation contexts. This effort was complemented by standardizing datasets, requiring local teams to use a FOSS Database Management System and a compatible data model. At one location, the local health commissioner wanted reports quickly, but the timeframes were too short for the local team to follow the FOSS and data model guidelines. The local team imported data into Excel, which they knew better, to create timely reports.

This departure from standards reflects contradictions of local and translocal institutional logics, creating a solution trap. The local team uses established practices (Excel for reporting), aligning with their institutional logic. They better meet local health needs and provide timely reports. The intervention (ie, standardized datasets) carries translocal institutional logics that favor standardization for national-level decision-making and international scalability. Furthermore, the intervention requires adapting practices without immediate benefits for local teams. So, the local team avoids standards misaligned with their institutional logic. The misalignment forces decisions between mutually exclusive solutions. They chose established practices because of direct accountability to the local health commissioner rather than vague accountability to the academic group. This generated technical debt that proved difficult to recover later.

#### Illustration 3: A Path Dependency Problem

This is even more prominent if, for instance, the use of digital tools in community-based health care could further amplify the solution trap. In community-based health care, CHWs provide basic health services to their community. Digital tools compensate for limited formal medical education by providing expertise remotely and across contexts. Medical algorithms ensure patient safety by prescribing appropriate procedures. CHWs follow these algorithms and interact with patients (eg, taking blood samples). Again, a single-minded solution approach seems reasonable, as studies have shown its feasibility. However, we wonder if such interventions are double-edged swords. Undoubtedly, CHWs are crucial to achieving Sustainable Development Goal 3 (health equity) by extending the health system’s reach into rural areas. However, we question how laypeople differentiate HIV rashes from other causes (eg, contact allergies). Digital tools might act as blinders, reducing the field of vision. This reduction in complexity enables CHWs to provide services they could not otherwise offer. Digital tools can empower CHWs to care for patients from prevention to treatment. Conversely, this approach creates strong dependencies on digital tools and implied actions.

### The Relevance of Solution Traps

In the most disparate situations in life, everyone happens to see that someone is doing something based on their knowledge of outcomes rather than being consequential on their premises. Our illustrations hint at an often-overlooked issue: targets of technological designs are not void solution recipients. Instead, they deal with several coexisting solutions for their problems. Consequently, new interventions add options to an already crowded solution space. Adding without considering removal complicates problems rather than solving them. We call these situations solution traps. They are particularly important to problematize in disciplines, such as DSR and medical informatics, which commonly assume the consequential behaviors that our examples above problematize [19,74]. We focus on their single-minded problem-solving, reducing targets to mere solution recipients. This orientation results in context blindness due to reductionism, used to control the complexity of the problem space.

Examining this incompatibility closely, the reason seldom lies in divergent motivations, values, or aims. The tension arises from the different prospective journeys (ie, different solution approaches). Often, stakeholders assume their solutions are easily transferable across contexts. Combined with an attempt to reduce complexity, this assumption results in inadequate consideration of established institutional logics and embedded technologies. Consequently, interventions are designed to change or replace existing behaviors and artifacts, even if they may undermine their stability and accountability lines because of interrelated practices. The friction between different institutional logics creates tensions, as illustrated above, that run deeper than typical organizational change problems (eg, resistance). The solution trap highlights misaligned institutional logics for existing and intended behaviors. The solution trap cannot be escaped by designing an artifact that simply promises to improve the existing behaviors without understanding what keeps them in place. Would patients benefit if personnel in the illustrations followed translocal logics?

In a nutshell, instead of spotting gaps in the literature, we problematize empirical observations [19,75] to identify common, yet overlooked solution traps, especially when it comes to “getting things done.” Although often outside designers’ mandate and scope, these situations can undermine their work. Thus, they warrant consideration. Our illustrations are not rare or extreme cases. Their peculiar global contexts highlight, not divert from, common solution traps. We propose a perspective that highlights the role of organizational contexts in providing different solutions, hindering coordination.

## Solution Patchwork

### Conceptualization

DSR methods help designers focus on users’ problems. Existing approaches provide valuable microlevel design tools. However, the artifact may not achieve the intended transformation if it is not adapted to the sociotechnical context. Practice research helps grasp user practices, serving as a starting point for understanding the underlying institutional logics. However, institutional logic is often hidden and unreflected, therefore difficult to elicit. Exposing users to the designer’s conceptualization of their behaviors and logic prompts reflection on behaviors and their institutional logic.

**Figure 1.**
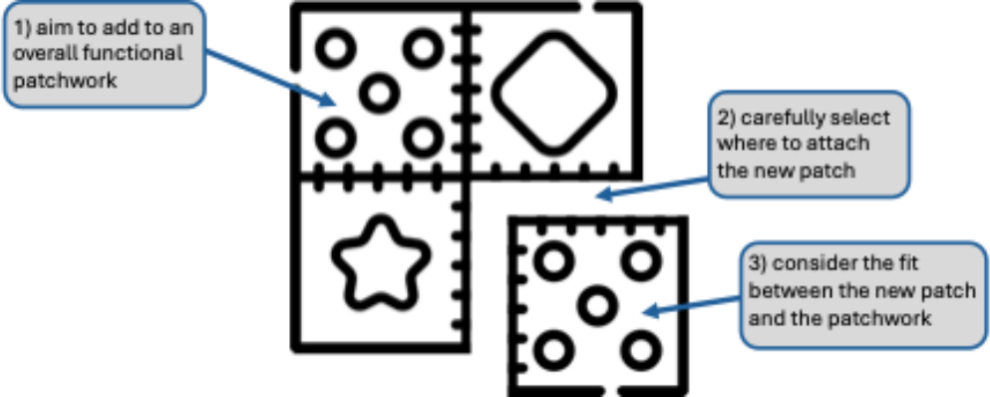
Solution patchwork concept.

We propose “solution patchwork” as a coordination approach to avoid solution traps. The *Cambridge Dictionary* defines patchwork as a “cloth consisting of smaller pieces of differently patterned cloth that are sewn together.” It is needlework where cloth pieces form a coherent pattern. Similarly, designers must integrate local and translocal aspects (which may not align with the micro and macro levels) when designing, deploying, and adjusting artifacts. When adding a patch, tailors decide on materials, color, and suitable stitching techniques when attaching it to its neighboring pieces. Designers face similar considerations. The patchwork combines established practices and artifacts—digital or analog. Like seams binding patches into a patchwork, institutional logics link practices, structures, and norms to form organizations, such as the health system in the Peruvian Amazon. Consequently, designers must understand institutional logics in their problem space to judge intended interventions. Only then can they ensure their new patch is compatible with the overall patchwork. A new patch may fit initially but disrupt the overall pattern. Solution patchwork emphasizes blending new artifacts with existing practices, not filling gaps or replacing dysfunctional practices. Designers must step back from the patchwork periodically to see the bigger picture. They might find empty spaces, overlapping patches, or damage from attaching another patch. Further, designers must also step back to avoid oversimplifications, which they often use to address the complexity of their design problems. Moreover, designers must constantly move between the micro and macro levels to create a coherent patchwork. Instead of designing to fill a void or improve the situation with a better solution, designing for institutional logics has three consequences (Figure 1):

1. The designer’s goal should be to add to an overall functional patchwork. Awareness of institutional logics enables them to better position their artifacts within the specific sociotechnical context. Their focus should shift from designing for individual practices to designing for broader contexts (including institutional logics) to support a successful transformation.
2. Designers should carefully select where they attach their new patch. Understanding current institutional logics (eg, diagnosing malaria because a remedy is available) enables designers to anticipate how their artifact may create friction with the context. They must find entry points that complement rather than compete with existing practices. In our illustration, medication availability might have guided the diagnosis. A possible entry point could be improving access to other medications, allowing doctors to provide more appropriate care.
3. Designers should consider the fit between their patch and the patchwork. The patchwork analogy might suggest that anything can be stitched together. However, combining colors and fabric types is central to the overall result. While we do not question the rigor of biomedicine, we highlight the need to adapt translocal solutions to local contexts. For example, biomedicine often starkly contrasts with traditional medicine. Those exposed to traditional medicine may be skeptical of or may reject biomedicine. Instead of replacing local health practices, embedding biomedicine within them could increase its acceptance. Similarly, theoretical concepts, such as empowerment, are based on specific cultural features. Recent studies report counterintuitive outcomes when transferred to other contexts [63].

### An Illustration of the Solution Patchwork

The solution patchwork could have been applied to our 3 solution trap illustrations. In Table 1, we exemplify the consequences of the solution patchwork on our third illustration “A Path Dependency Problem.”

**Table 1.** Applying solution patchwork: principles, design questions, and illustration in community-based care.

Solution patchwork principle	Design question	Application in the CHW^a^ illustration	Expected benefit
Aim to add to an overall functional patchwork	How does the proposed artifact fit within the broader sociotechnical context rather than only improving an isolated task?	Before deployment, the design team maps the wider context of CHW work, including local authorities, community expectations, reliance on traditional medicine, and supply realities. The tool is positioned as support for CHWs’ real decision-making constraints rather than as a replacement for existing practices.	Sensitizes designers to the relevant components of the existing sociotechnical context and helps them define the intervention in relation to that broader structure rather than as a stand-alone fix.
Carefully select where to attach the new patch	At which points can the artifact complement, rather than compete with, existing practices and workflows?	Designers identify attachment points that strengthen existing referral structures. Instead of inserting isolated diagnostic pathways, they add prompts for clinician consultation and cues to distinguish common rashes, aligned with CHWs’ knowledge and routines.	Helps designers identify feasible entry points for intervention and anticipate, where friction, duplication, or contradiction with existing practices may arise.
Ensure a good fit between patch and patchwork	Does the artifact align with local explanatory models, norms, and expectations, or does it distort the wider pattern of practice?	Designers refine the tool so that its logic aligns with how CHWs and trained clinicians understand health. For example, when a rash may have non-HIV causes, CHWs could photograph it for image-based assessment or clinician review.	Encourages designers to assess compatibility between the new artifact and the wider configuration of practices, meanings, and accountabilities, increasing the likelihood of coherent integration.

a CHW: community health worker.

## Discussion

### Principal Findings

As argued, the root cause of the solution trap often stems not from misaligned motivations or values among stakeholders, but from the incompatibility of coexisting solutions. In illustration 1, a telemedicine system based on biomedical diagnose-then-treat logic collided with local pressures to maintain stability under scarcity, so diagnoses were shaped by drug availability rather than the digital tool’s intended logic. In illustration 2, the shift toward standardized open-source infrastructure clashed with local reporting practices and accountability requirements, leading teams to revert to Excel despite global pushes for scalability. In illustration 3, algorithmic support for CHWs promised safer care through standardization yet simultaneously increased digital dependency and narrowed diagnostic vision. Together, these cases show that what appears to be a lack of solutions is actually a landscape of overlapping systems. The tension between these incompatible approaches produces the solution trap—a pattern requiring further contextualization within existing research.

### Differentiation From Related Concepts

Several concepts relate to both the solution trap and solution patchwork, sharing some but not all characteristics. In Table 2, we address selected overlapping ideas and clarify their core differences from our specific conceptualizations. This approach ensures precision and provides readers with useful lenses for examining the problems mentioned above.

**Table 2.** Related concepts and how they differ from the solution trap and solution patchwork.

Related concept	Core idea	Overlap with solution trap or solution patchwork	Key difference	Relevance for this manuscript
Design-reality gap	Failure emerges when design assumptions do not match realities on the ground [2,76].	Both perspectives emphasize context and explain why interventions fail when local conditions are insufficiently understood.	The design-reality gap focuses on mismatch between design and reality; the solution trap focuses on incompatibility among multiple coexisting solutions already operating in the context.	Shows that the issue is not only poor fit of one intervention but also conflict with existing sociotechnical arrangements.
Installed base	New infrastructures must evolve from existing sociotechnical arrangements that both enable and constrain change [77].	Both recognize that existing arrangements shape what new interventions can do.	Installed base emphasizes cultivation and gradual evolution of infrastructure; solution patchwork emphasizes coordination among overlapping solutions and their institutional logics.	Clarifies that the manuscript is not only about infrastructural inertia, but about how to attach new “patches” without intensifying conflict.
Functionalism	Researchers simplify complex situations to make problems manageable and interventions designable [78,79].	Both acknowledge that practical action requires some reduction of complexity and attention to what works in practice.	The manuscript critiques excessive simplification that creates a false “void” in need of a solution; solution patchwork instead calls for reflexive attention to existing practices, their functions, and their unintended consequences [80-82].	Positions the argument against decontextualized problem framing while retaining a practical orientation toward action.
Structuration theory	Technologies, actors, and structures mutually shape one another over time [83].	Both perspectives reject purely technical accounts and emphasize embeddedness in broader social arrangements.	Structuration theory offers a broad theoretical account of mutual shaping; solution trap and solution patchwork translate similar concerns into more accessible concepts for design and medical informatics practice.	Helps connect the argument to established sociotechnical theory while showing the added value of a more practice-oriented heuristic.
Jugaad or bricolage	Actors recombine available resources creatively to address local needs under constraints [84,85].	Both value bottom-up adaptation, resource recombination, and sensitivity to situated practice.	Jugaad and bricolage foreground improvisation; solution patchwork adds explicit attention to institutional logics and to the compatibility of new and existing sociotechnical elements.	Shows that the manuscript builds on bottom-up innovation traditions but adds a coordination lens for complex health systems.

### Implications for Medical Informatics Research and Practice

While several conceptualizations attempt to capture the complexity of uneven contexts, practical approaches for addressing these challenges in medical informatics research remain scarce. Building on existing frameworks and our own findings, we provide the following guidance. We propose that design-oriented projects in digital health should begin by recognizing the existing solutions, each backed by its own institutional logics, infrastructures, and practices. This implies expanding the problem-space exploration to systematically identify existing solutions, trace how they coexist and interact, and surface the institutional logics that make some combinations workable and others conflictual, before locking in a problem framing and design trajectory.

In medical informatics, the solution trap narrows the focus to designing increasingly sophisticated digital tools. The uncertain real-world impact of many rigorously evaluated digital health artifacts—and the low uptake of tools that excel in controlled studies—arises from ignoring how new interventions interact with existing solutions within uneven health systems. Rather than simply asking whether a new telemedicine platform, decision support tool, or data infrastructure works in isolation, medical informatics projects must examine how these solutions reconfigure workflows, redistribute accountabilities, and either intensify or ease tensions between local and translocal institutional logics. This requires foregrounding the practices of health workers and patients, including their entrenched workarounds and informal infrastructures, as solutions in their own right rather than as mere obstacles to the deployment of a new artifact.

The concept of solution patchwork offers a practical heuristic for DSR and medical informatics to navigate these challenges. By viewing the target context as a collection of coexisting solutions, designers must decide which new patch to develop, where to attach it, and which existing pieces to align or leave untouched. Furthermore, they must treat institutional logics as the seams that hold—or tear—the patchwork together. In concrete terms, this implies designing for combination rather than replacement, articulating how proposed artifacts connect to, delegate to, or deliberately sidestep existing solutions, and evaluating success in terms of how the overall patchwork functions under different sociotechnical conditions rather than only in terms of single-solution performance metrics. By shifting focus from filling perceived gaps to integrating overlapping solutions, DSR and medical informatics can avoid solution traps. This approach strengthens the resilience of digital health interventions, especially in globally uneven settings.

The notions of the solution trap and solution patchwork serve as useful tools throughout a design project. In the initial phase, they support systematic analysis of existing solutions to identify established components. During design and development, they help ensure new solutions align with institutional logics and other contextual characteristics. In evaluation, they offer a broader perspective that extends beyond traditional measurement-oriented designs. We acknowledge that applying these notions in practice requires further exploration. Future research could substantiate the solution patchwork approach in 2 ways. First, more empirical case studies from digital health and other fields would enhance generalizability. Second, integrating solution patchwork into different problem-solving methods would strengthen its validity, clarifying how this context-centric approach differs from problem-, solution-, or system-centric models.

## Abbreviations

AI: artificial intelligence
CHW: community health worker
DSR: design science research
